# Targeting endothelial connexin40 inhibits tumor growth by reducing angiogenesis and improving vessel perfusion

**DOI:** 10.18632/oncotarget.7370

**Published:** 2016-02-13

**Authors:** Florian Alonso, Sonia Domingos-Pereira, Loïc Le Gal, Laurent Derré, Paolo Meda, Patrice Jichlinski, Denise Nardelli-Haefliger, Jacques-Antoine Haefliger

**Affiliations:** ^1^ Department of Medicine, Lausanne University Hospital, Lausanne, Switzerland; ^2^ Department of Urology, Lausanne University Hospital, Lausanne, Switzerland; ^3^ Department of Cell Physiology and Metabolism, University of Geneva, Medical Center, Geneva, Switzerland

**Keywords:** cell-cell communication, connexins, angiogenesis, tumors, transgenic mice

## Abstract

Endothelial connexin40 (Cx40) contributes to regulate the structure and function of vessels. We have examined whether the protein also modulates the altered growth of vessels in tumor models established in control mice (WT), mice lacking Cx40 (Cx40−/−), and mice expressing the protein solely in endothelial cells (Tie2-Cx40). Tumoral angiogenesis and growth were reduced, whereas vessel perfusion, smooth muscle cell (SMC) coverage and animal survival were increased in Cx40−/− but not Tie2-Cx40 mice, revealing a critical involvement of endothelial Cx40 in transformed tissues independently of the hypertensive status of Cx40−/− mice. As a result, Cx40−/− mice bearing tumors survived significantly longer than corresponding controls, including after a cytotoxic administration. Comparable observations were made in WT mice injected with a peptide targeting Cx40, supporting the Cx40 involvement. This involvement was further confirmed in the absence of Cx40 or by peptide-inhibition of this connexin in aorta-sprouting, matrigel plug and SMC migration assays, and associated with a decreased expression of the phosphorylated form of endothelial nitric oxide synthase. The data identify Cx40 as a potential novel target in cancer treatment.

## INTRODUCTION

Angiogenesis, i.e. the growth of new capillaries sprouting from pre-existing blood vessels, is fundamental to many physiological and pathological events, including tumor survival and growth [1]. The mechanism leading to angiogenesis requires the coordinated response of endothelial cells (ECs) to multiple stimuli [2], including basic fibroblast growth factor, vascular endothelial growth factor and nitric oxide (NO), which stimulate the proliferation, survival, migration and differentiation of ECs [3–5]. As yet, however, such molecules have shown modest and transient effects in cancer therapy [6–8], thus calling for the identification of novel endothelial targets.

Connexins (Cx) form channels for the electrical and metabolic signaling, which coordinates the functions of individual cells [9], notably within the wall of normal blood vessels [10–12]. Between ECs, this signaling is mediated by Cx40 and Cx37 [13]. The combined invalidation of Cx40 and Cx37 causes lethal, perinatal hemorrhages, suggesting that these 2 proteins are required for the normal development of the microvasculature [14]. Cx40-null mice (Cx40−/−) also display impaired endothelial-dependent dilation of blood-vessels in response to various agents [15, 16], and are hypertensive mainly due to increased renin secretion [17–19].

We hypothesized that Cx40 may also participate in the angiogenic response of tumoral tissues. To test this hypothesis, we have compared WT mice, expressing control levels of Cx40, with Cx40−/− mice, which lack this Cx [20] and with Tie2-Cx40 animals, which specifically express Cx40 solely in ECs [21]. We have further studied the *in vitro* and *in vivo* effect of a peptide which mimics extracellular portions of Cx40, and specifically targets this protein [22, 23]. The data provide direct evidence that Cx40 plays a pivotal role in the control of angiogenesis and tumor growth, possibly by reducing NO production.

## RESULTS

### Loss of endothelial Cx40 decreases tumor growth and vessels, independently of hypertension

**Figure 1:**
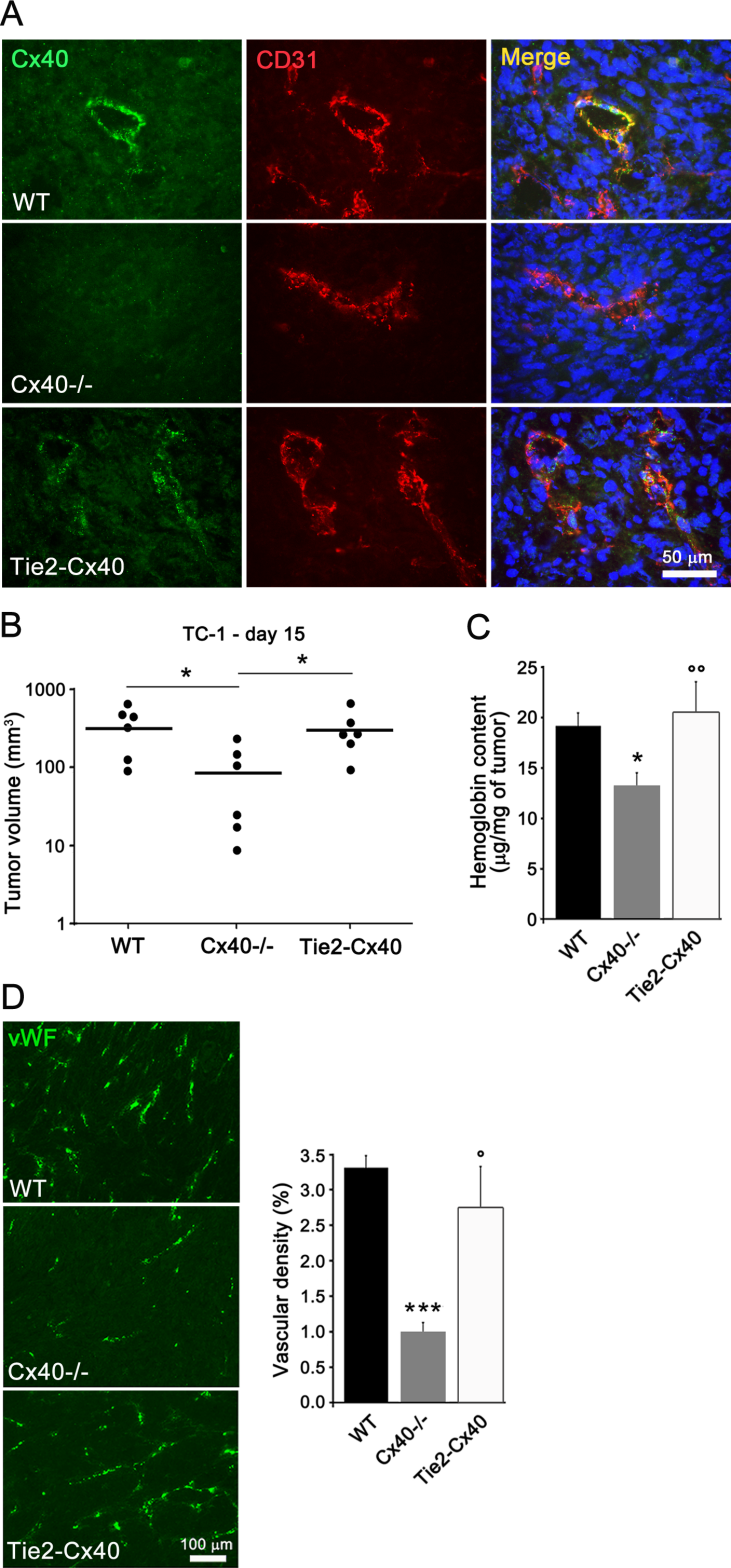
Loss of Cx40 decreases the growth and angiogenesis of TC-1 s.c. tumors (**A**) Immunostaining of Cx40 showed the presence of the connexin at ECs (identified by co-staining for CD31) of tumors which grew in WT and Tie-2-Cx40 mice, but not in those which developed in Cx40−/− mice. (**B**) 14 days after the injection of TC-1 cells, Cx40−/− mice develop s.c tumors of smaller size compared to WT and to Tie2-Cx40. Dots represent individual mice; horizontal lines show geometric mean values. (**C**) The normalized hemoglobin content was also similar in the tumors of the Tie2-Cx40 and WT mice, but lower in the tumors of Cx40−/− mice. Data are mean + SEM of 6 mice per group. (**D**) Immunostaining of the endothelial specific von Willebrand (vW) factor revealed a similar density of vessels in the TC-1 tumors which developed in the Tie2-Cx40 and WT mice, and much lower in the tumors that developed in Cx40−/− mice. Data are mean + SEM of 6–7 areas from tumors of similar size that developed in 5 different mice per group. Significant differences are shown as *p < 0.05; **p < 0.01; ***p < 0.001 versus WT mice and °*p* < 0.05; °°*p* < 0.01 versus Cx40−/− mice.

We compared knock-out mice which lack Cx40 and transgenic Tie2-Cx40 mice [21], in which this protein is solely restored in ECs (Figure 1A). Two weeks after the implantation of TC-1 cells into the flank of either WT, Cx40−/− or Tie2-Cx40 mice, sizable tumors with capillaries expressing Cx40 (Figure 1A) had grown in all WT and Tie2-Cx40 mice (Figure 1B). In contrast, smaller tumors had grown in Cx40−/− mice (Figure 1B), which exhibited a lower hemoglobin content and density of blood vessels than the tumors of WT and Tie2-Cx40 animals (Figure 1C–1D). The data document that the loss of endothelial Cx40 reduces growth and angiogenesis of a subcutaneous (s.c.) tumor.

**Figure 2:**
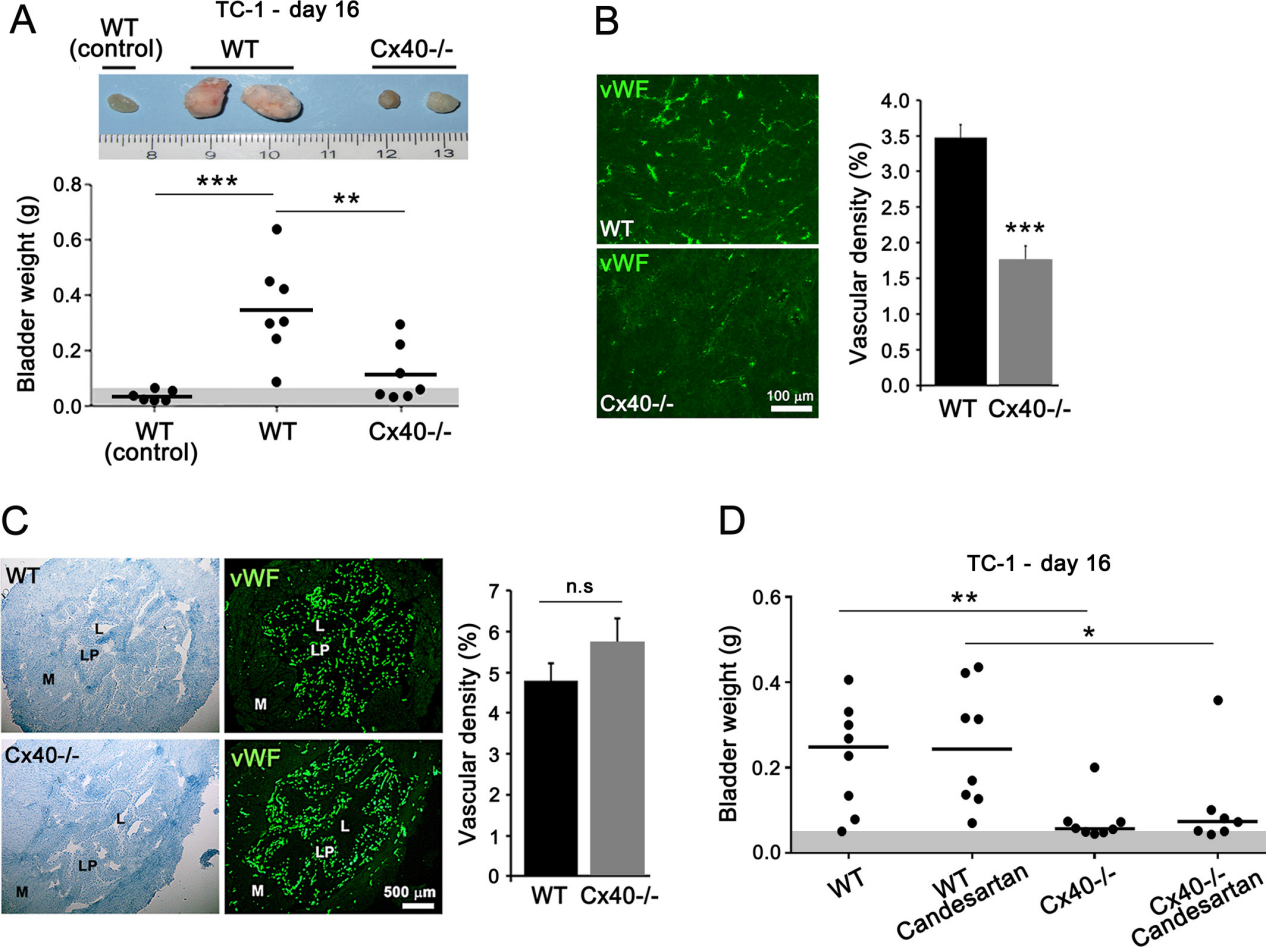
Loss of Cx40 decreases growth and angiogenesis independently of hypertension in bladder-tumors (**A**) In WT mice, the size (top) and weight of the bladders (bottom) was higher in the animals instilled with TC-1 cells than in non instilled controls. TC-1-instilled Cx40−/− mice featured bladders with sizes and weights significantly smaller than those seen in the corresponding WT mice, Dots represent individual mice; horizontal lines show mean values. Gray area represents the mean weight + 3 SD of native mice bladders. (**B**) Immunostaining for vWF revealed a lower density of vessels in the TC-1 tumors which developed in the bladders of Cx40−/− than of WT mice. Data are means + SEM of 4–7 areas from similar size tumors in 3–4 different mice per group. (**C**) Immunostaining of vWF revealed a similar density of vessels in the native bladder of WT and Cx40−/− mice. Data of right panel are mean + SEM values of 3 fields, photographed in each of 3 mice per group. (**D**) The difference in the weights of bladders between WT and Cx40−/− mice instilled with TC-1 cells, was not altered by the Candesartan treatment. Dots represent individual mice; horizontal lines show mean values. Significant differences are shown as *p < 0.05; **p < 0.01; ***p < 0.001 versus respective WT mice.

Comparable observations were made after the instillation of TC-1 cells in the bladder of Cx40−/− mice (Figure 2A and 2B). In contrast, the native bladders of both WT and Cx40−/− mice featured a similar network of capillaries (Figure 2C), irrespective of the absence (Cx40−/−) or presence (WT; Supplementary Figure S1) of EC Cx40. The data show that loss of this protein only alters the growth and the angiogenesis of transformed tissues.

Given that Cx40−/− mice are hypertensive [17, 19] and that angiogenesis may be influenced by blood pressure [24], the experiments were repeated using mice treated with Candesartan for 3 weeks. The drug decreased to control levels the blood pressure of Cx40−/− mice, without affecting the normal blood pressure of WT controls (Supplementary Figure S2). Under these conditions, the Candesartan-treated WT-mice developed bladder-tumors similar to those of untreated controls (Figure 2D), whereas Candesartan-treated Cx40−/− mice developed fewer and smaller tumors, similar to those of the untreated hypertensive companions (Figure 2D). The data show that loss of Cx40 decreases the growth and angiogenesis of bladder tumors by a mechanism which is not affected by blood pressure.

### Loss and inhibition of Cx40 decrease angiogenesis

**Figure 3:**
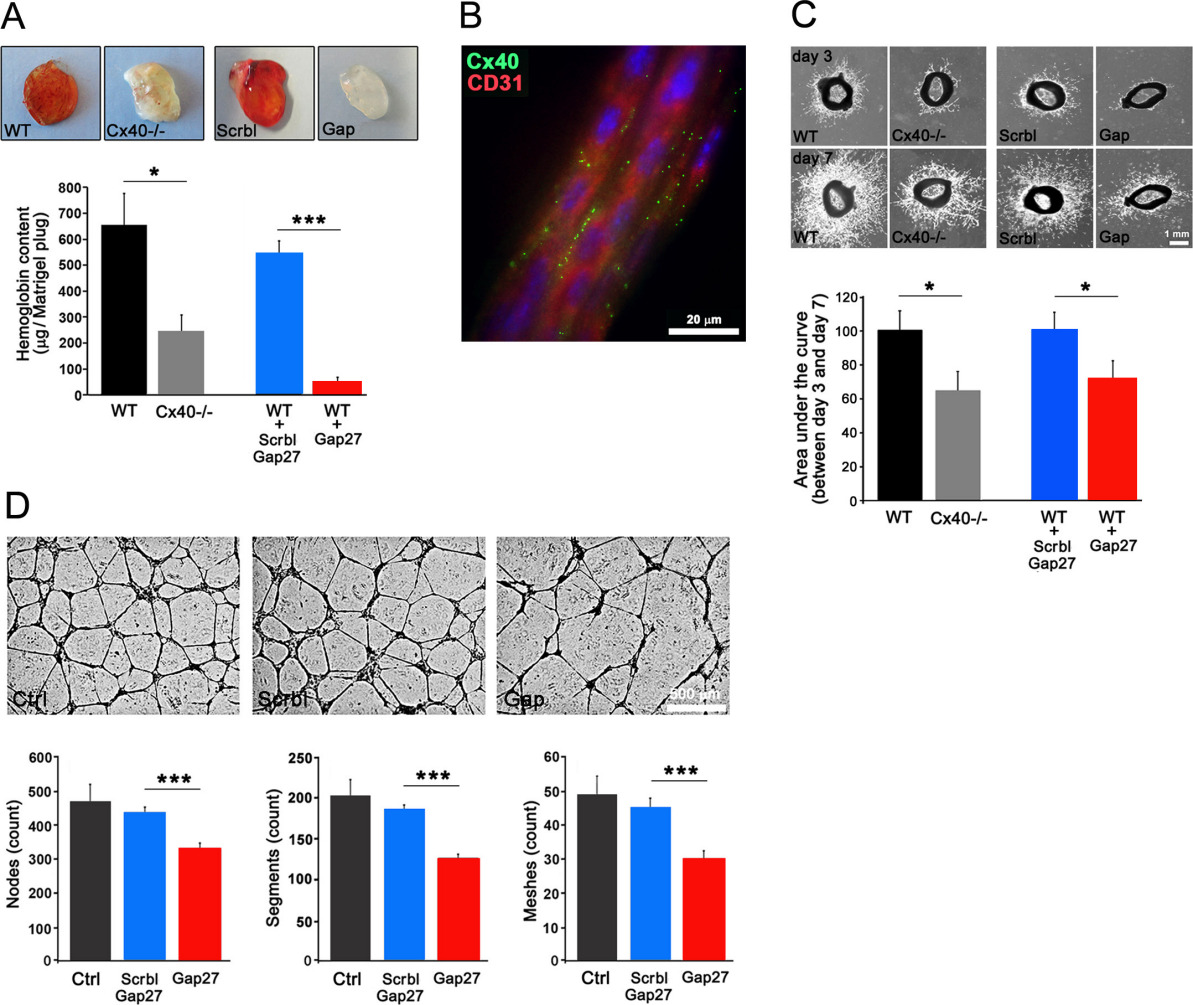
Loss of Cx40 attenuates angiogenic potential of ECs (**A**) One week after the s.c. injection of matrigel plugs supplemented with VEGF, both the red color and the hemoglobin content were found reduced in plugs retrieved from Cx40−/− mice compared to WT mice. In WT mice, VEGF matrigel plugs supplemented with a scrambled version of the ^40^Gap27 peptide resembled the plugs containing only VEGF (WT). In contrast, in matrigel plugs prepared with VEGF and the ^40^Gap27 peptide, both the red color and the hemoglobin content were sizably reduced, and resembled those observed in Cx40−/− mice. Data are mean + SEM of 9–15 mice per group. (**B**) After a 7 day culture, immunostaining of vessels sprouting out from aortic rings shows the spotted distribution of Cx40 (green spots) on CD31-identified ECs (red). (**C**) After a 3 day culture, aortic rings of WT mice show a sizable capillary outgrowth, which increased with culture time. This sprouting was reduced in aortic rings of Cx40−/− mice or of WT mice in the presence of the ^40^Gap27 peptide, which targets Cx40. Data are mean + SEM of 10–15 rings from 3–4 mice per group. (**D**) *In vitro*, the ability of HUVECS to form capillary-like structures onto matrigel was altered in presence of the ^40^Gap27 peptide. Results are means + SEM from 3 separate experiments performed in triplicate and 2 different fields per replicate were pictured.

One week after the s.c. implantation of matrigel-plugs supplemented with heparin and Vascular Endothelial Growth Factor (VEGF), an intense red staining and sizable hemoglobin content of the plugs, indicating extensive angiogenesis, was observed in WT mice (Figure 3A). Both parameters were significantly reduced in the plugs implanted in Cx40−/− mice (Figure 3A). Parallel experiments revealed that supplementing the plugs with ^40^Gap27, a peptide known to specifically inhibit Cx40 function [22, 23, 25], reduced the angiogenesis in WT mice, in contrast to plugs supplemented with a scrambled version of this peptide (Figure 3A). Comparable observations were made with matrigel plugs containing Fibroblast Growth Factor (FGF) (Supplementary Figure S3). The data demonstrate that interfering with Cx40 function reduces the *in vivo* neo-angiogenesis, whichever the angiogenic stimulus tested. This was further confirmed by the absence of Vascular Endothelial Growth Factor Receptor-2 modulation in TC-1 s.c tumors of Cx40−/− mice (Supplementary Figure S4).

After 4 days of culture in matrigel, aorta explants from WT mice showed sprouting of capillary-like structures, whose ECs co-expressed Cx40 and CD31 (Figure 3B). This sprouting, which increased for the 7-day duration of the experiment (Figure 3C), was significantly reduced in explants from both Cx40−/− mice and from WT mice exposed to the ^40^Gap27 peptide (Figure 3C).

In another set of experiments, primary human ECs (HUVECs), were seeded onto matrigel, to form vascular-like 3D structures [26]. The development and branching of these structures, were significantly reduced by addition of ^40^Gap27, but not its scrambled form (Figure 3D). The data document that Cx40 increases EC migration, vessel sprouting and growth.

### The absence of Cx40 improves the perfusion and function of tumor-vessels

**Figure 4:**
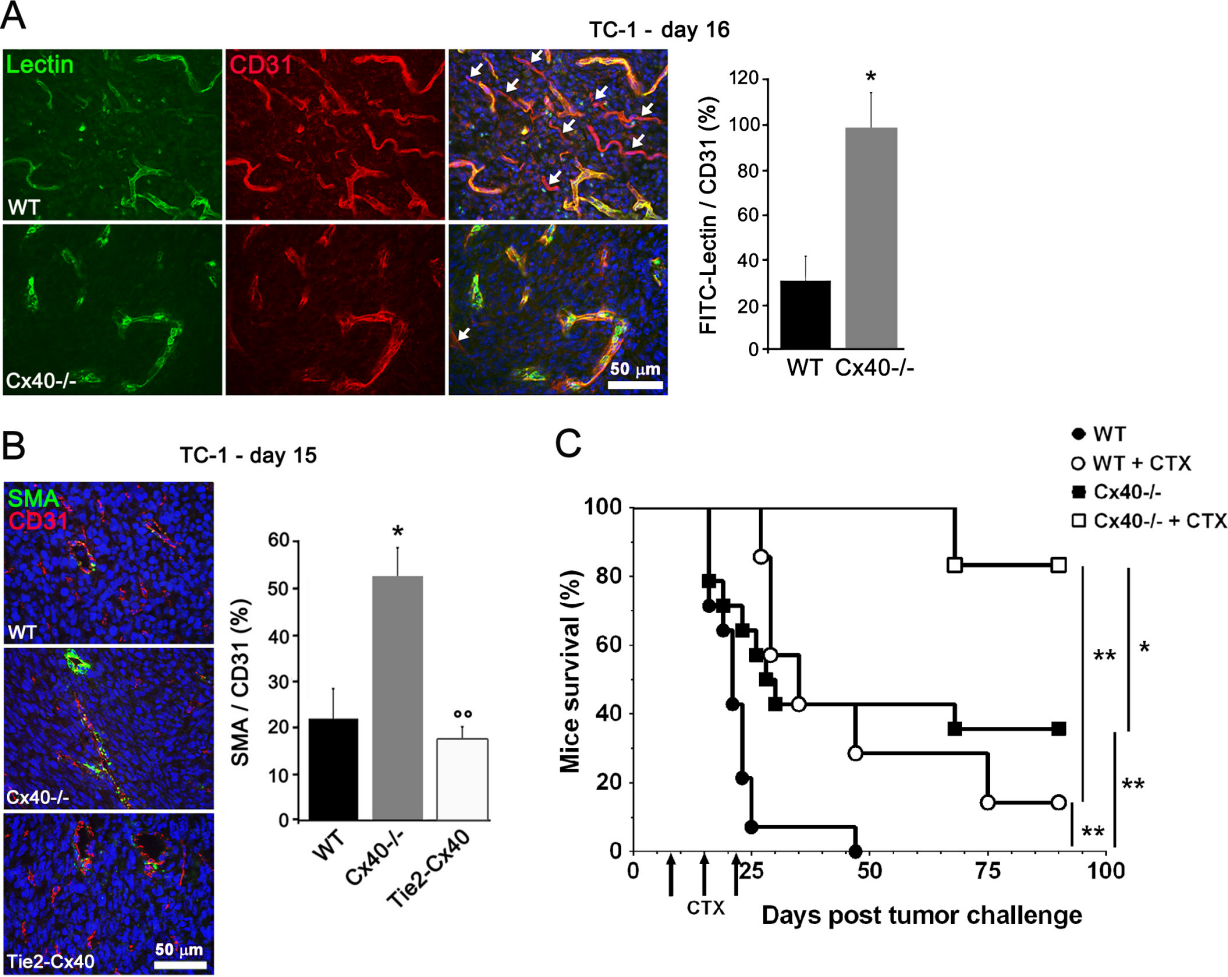
Cx40 deficiency causes enhanced tumor vessel maturation and functionality (**A**) Tomato lectin (green) labeled most of the vessels (identified in red by immunostaining for CD31) in tumors grown in Cx40−/− whereas some vessels were not stained by lectin in WT mice; white arrows indicate tumor vessels (CD31+) that were not perfused (lectin-). Data are mean + SEM of 8–10 fields from 3 different mice per group. (**B**) Immunostaining of alpha-SMA and CD31 showed the distribution of SMCs and ECs in the vessels of TC-1 tumors. The density of co-stained vessels was higher in the tumors induced in Cx40−/− than in those induced in Tie2-Cx40 and WT mice. Data are mean + SEM of 3–8 fields from 4–5 different mice per group. *p < 0.05 versus WT mice and °°*p* < 0.01 versus Cx40−/− mice. (**C**) Cyclophosphamide (CTX) treatment (arrows) of WT mice s.c. injected with TC-1 (*n* = 7, open circles) significantly prolonged mice survival (14% of surviving mice at the end of the 90 days experiment), as compared to WT untreated mice that all died within 7 weeks (*n* = 14; solid circles, *p* < 0.01). Survival of Cx40−/− mice injected with the same amount of TC-1 cells (*n* = 14; solid squares) was significantly higher than WT mice (36% of surviving mice, *p* < 0.01). Even more effective, CTX treatment in Cx40−/− mice (*n* = 6; open squares) resulted in 83% of the mice surviving (*p* < 0.01 as compared to CTX-treated WT mice). Significant differences by a log rank test are shown *p < 0.05 or **p < 0.01.

After i.v. injection of FITC-labeled tomato lectin in tumor-bearing mice, we found that more vessels were labeled by the lectin in tumors grown in Cx40−/− than WT mice (Figure 4A), indicating increased perfusion of tumor vessels lacking Cx40. Immunostaining for smooth-muscle actin (SMA), further revealed more mural cells around the vessels of tumors established in Cx40−/− than in WT mice (Figure 4B). This increase was associated with the loss of endothelial Cx40, inasmuch as it was not observed in the vessels of tumors grown in Tie2-Cx40 mice (Figure 4B). The data demonstrate that the absence of Cx40 improved the function and structure of tumor vessels.

Seven, 14 and 21 days after the s.c. implantation of TC-1 cells, we i.p. injected WT and Cx40−/− mice with cyclophosphamide (CTX). In WT mice, this treatment prolonged mice survival (14% of surviving mice at the end of the 90 days experiment), as compared to that observed for untreated WT mice, which all died within 7 weeks (Figure 4C). Under the same conditions, the survival of Cx40−/− mice was significantly longer (83% and 36% of the mice surviving at the end of the experiment, in the presence and absence of CTX administration, respectively (Figure 4C). The data show that lack of Cx40−/− extends the survival of tumor-bearing mice, especially in the presence of a cytotoxic drug.

### Inhibition of EC Cx40 promotes the recruitment of vascular SMCs and decreases eNOS activation

**Figure 5:**
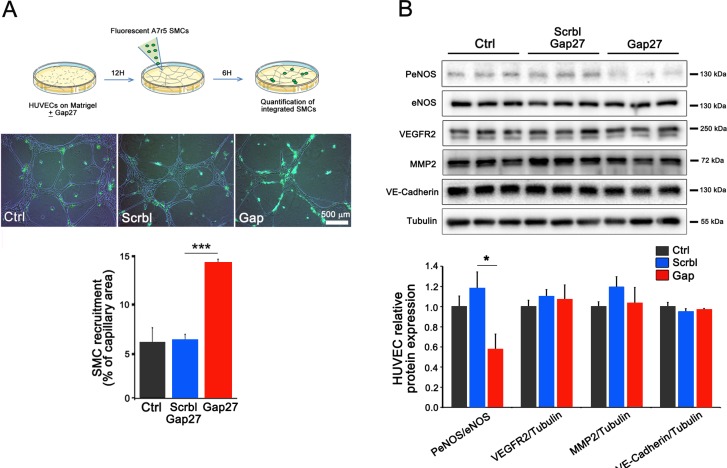
Cx40 inhibition in ECs promotes the recruitment of vascular SMCs (**A**) Fluorescent A7r5 SMCs were added in wells containing a capillary network of HUVECs. After 6 hours, quantitative assessment of the number of attached SMCs revealed an increased recruitment of A7r5 cells when the capillary-like structures were generated under the presence of the ^40^Gap27 peptide compared to its scrambled version. Results are means + SEM from 3 different experiments performed in duplicate. 2–3 pictures per replicate have been analyzed. (**B**) Western blot analysis performed on 2D HUVECs revealed that Cx40 inhibition during 4 hours using the ^40^Gap27 peptide reduces the phosphorylation of eNOS whereas, VEGFR2, MMP2 and VE-cadherin were unaffected. Data shown are means + SEM from 3 separate experiments performed in triplicate. Significant differences are shown as *p < 0.05; ***p < 0.001 versus Scramble peptide treatment.

In the presence of ^40^Gap27, an increased recruitment of A7r5 SMCs to HUVEC-made vessels was observed (Figure 5A), suggesting that Cx40 signaling negatively regulates SMC and/or stromal recruitment to newly formed vessels. Western blots further showed that a 4 h targeting of Cx40 using ^40^Gap27, reduced the expression of PeNOS, as compared to the levels observed in both untreated cells and cells exposed to the scrambled peptide, while the expression of other angiogenesis-related proteins, such as VEGFR2, MMP2, VE-cadherin and eNOS was unchanged (Figure 5B). The data document that Cx40 decreases eNOS signaling in ECs.

### Loss of Cx40 decreases the growth and angiogenesis of B16-F10 tumors and reduces PeNOS expression

**Figure 6:**
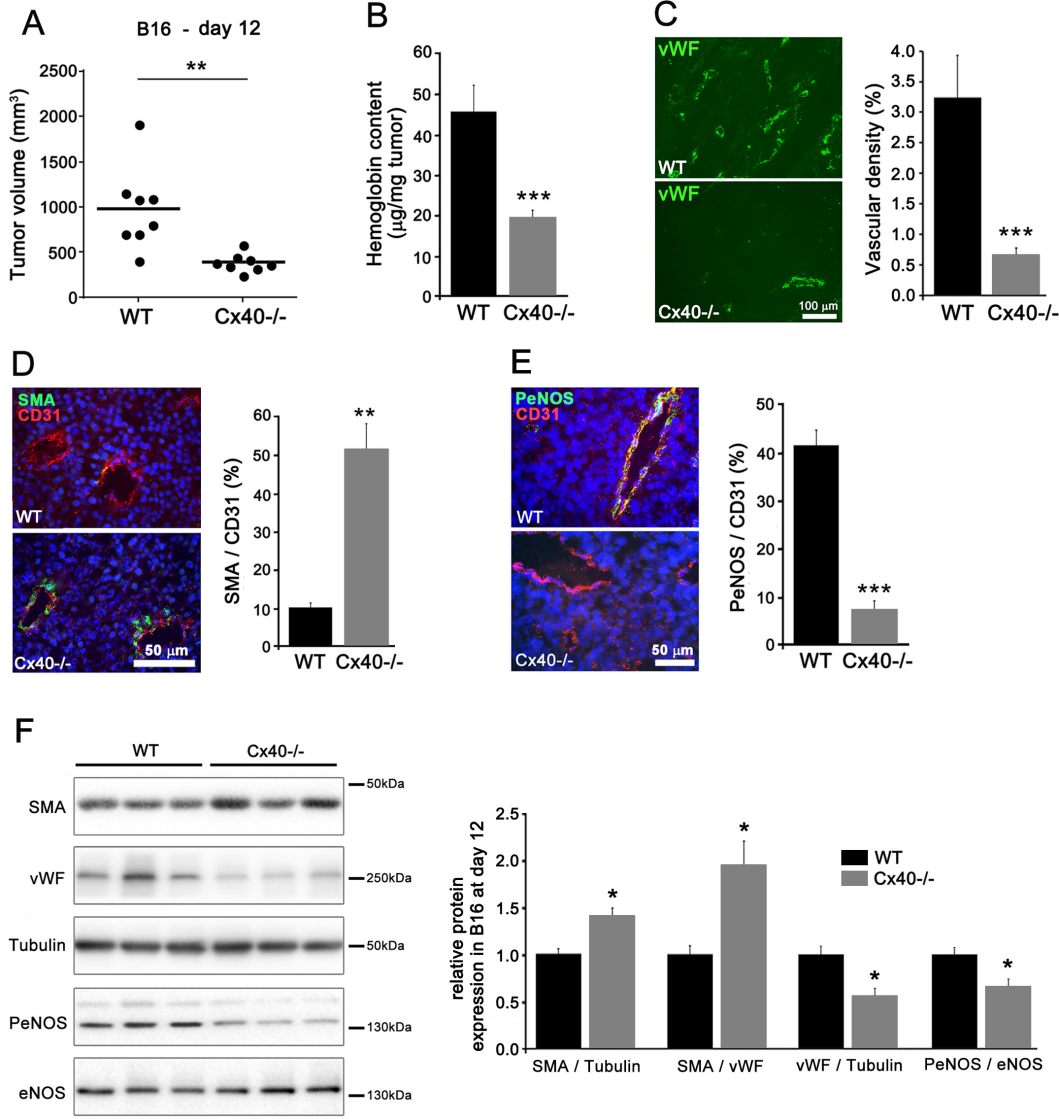
Loss of Cx40 attenuates the growth and angiogenesis of subcutaneous B16-F10 tumors (**A**) 11 days after the s.c. injection of B16-F10 cells, tumors of smaller size developed in Cx40−/− mice than in WT mice (*p* < 0.01). Dots represent individual mice; horizontal lines show mean values. (**B**) The normalized hemoglobin content was also lower in Cx40−/− tumors. Data are mean + SEM of 8 mice per group (*p* < 0.001). (**C**) Immunostaining of vWF revealed a lower density of vessels in the B16-F10 tumors grown in Cx40−/− than in WT mice (*p* < 0.001). Data are mean + SEM of 3–4 areas from 3 different mice per group. (**D**) Immunostaining of SMA and CD31 showed the distribution of SMCs and ECs in the vessels of B16 tumors. The volume density of SMCs was higher in the tumors induced in Cx40−/− than in those induced in WT mice (*p* < 0.01). Bar, 50 μm. Data are mean + SEM of 3–9 fields from 3 different mice per group. (**E**) Immunostaining showed the presence of PeNOS in the CD31 positive ECs of the B16 tumors grown in WT mice. The staining for the former molecule was lower in the tumors induced in Cx40−/− than in WT mice (*p* < 0.001). Data are mean + SEM of 3–6 fields from 3 different mice per group. (**F**) Western blots demonstrated a significant increase in SMA/vWF and SMA/Tubulin (*p* < 0.05) and a significant decrease in in vWF/Tubulin and PeNOS/eNOS (*p* < 0.05) in tumors grown in Cx40−/− mice. Data are mean + SEM of 6 different mice per group. Significant differences are shown as *p < 0.05.

Eleven days after the s.c. implantation of B16-F10 melanoma cells, sizable tumors had grown in all WT mice (Figure 6A). In contrast, Cx40−/− mice developed significantly smaller tumors (Figure 6A), which featured a lower hemoglobin content (Figure 6B) and vascular density (Figure 6C), but higher levels of SMA (Figure 6D). In addition, the expression of PeNOS was significantly decreased in the vessels of B16 tumors grown in Cx40−/− mice, compared to the levels seen in WT mice (Figure 6E and 6F). Comparable observations were made in the TC-1 tumors (Supplementary Figure S5). The data show that, in the absence of Cx40, the modulation of angiogenesis and growth of different tumor models is associated to decreased PeNOS expression.

### Targeting Cx40 decreases tumoral angiogenesis and growth

**Figure 7:**
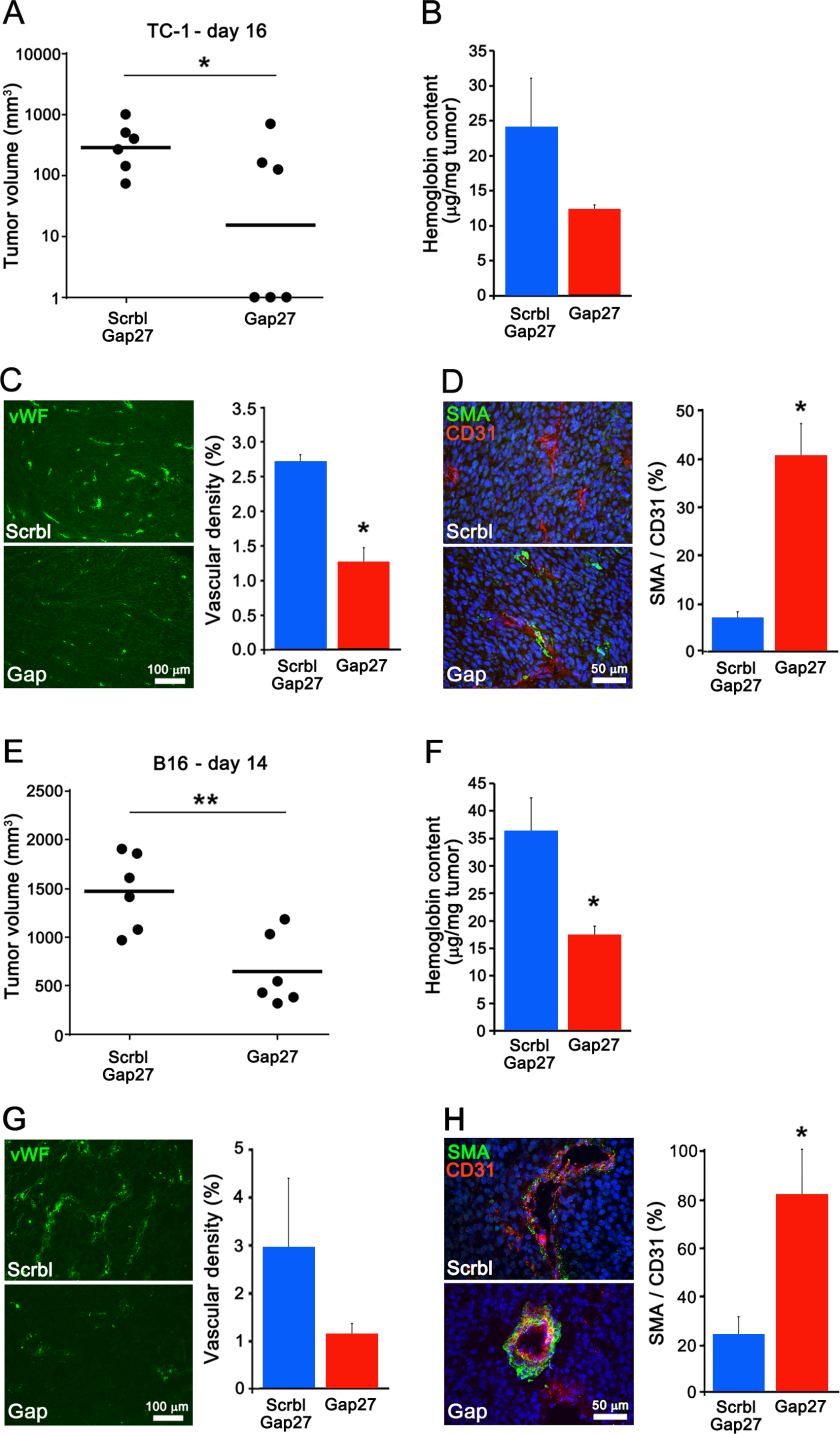
A peptide targeting Cx40 decreases tumoral growth and angiogenesis (**A** and **E**) Two weeks (15 and 13 days respectively) after the injection of WT mice with either TC-1 (A) or B16 cells (E), the volume of the resulting tumors was significantly lower in the mice which received ^40^Gap27, than in those which received the scrambled version of the peptide. Dots represent individual mice; horizontal lines show geometric (A) and arithmetic (E) mean values. (**B** and **F**) the hemoglobin content was also lower in the tumors grown in mice treated with ^40^Gap27 peptide than in those which received the scrambled version of the peptide. (**C** and **G**) Immunostaining of vWF revealed a lower density of vessels in the tumors grown in mice which received the ^40^Gap27 peptide. (**D** and **H**) Immunostaining of SMA revealed a higher density of SMCs in the tumors induced in mice treated with the ^40^Gap27 peptide. In all bar graphs, data are mean + SEM of 3–9 fields from 3 different mice per group. Significant differences are shown as *p < 0.05; **p < 0.01.

One day after the implantation of either TC-1 or B16 cells, mice were injected daily i.p. with 100 μg ^40^Gap27, which has been reported to inhibit Cx40, or its scrambled version. Within 2 weeks, all mice injected with ^40^Gap27 developed significantly fewer and smaller tumors (Figure 7A–7E), with a less developed vascularization (Figure 7B–7C–7F–7G) but an increased SMA coverage (Figure 7D–7H), than the mice injected with the scrambled peptide. The data show that targeting Cx40 with a mimetic peptide recapitulates the observations made in Cx40−/− mice.

## DISCUSSION

We report that Cx40, one of the prominent proteins that, together with Cx37 and Cx43, form gap junctions between ECs, participates to control angiogenesis in transformed tissues. Using WT, Cx40−/− and Tie2-Cx40 mice, as well as a peptide known to specifically target Cx40, we show that loss of Cx40 is associated with decreased angiogenesis in different *in vitro*, *ex vivo* and *in vivo* models. We further demonstrate that Cx40 participates to control the growth of melanoma and uro-genital tumors in association with changes of tumoral-vessels, and altered eNOS activity. In the absence of Cx40, the newly-formed tumoral vessels also formed an almost control wall structure, including a substantial coverage of the ECs by many SMCs, which resulted in an improved perfusion of the growing tumor, presumably accounting for the extended mouse survival observed after cyclophosphamide administration. These effects appear largely attributable to the Cx40 expressed within the vascular endothelium, inasmuch as they were prevented in transgenic Tie2-Cx40 mice in which Cx40 was exclusively restored in the ECs of Cx40−/− animals [21].

The mechanism(s) underlying the connexin effects remain(s) to be fully unraveled. Knocking down of Cx40 in HUVECs using specific siRNA, was already shown to decrease the formation of capillary-like structures [27]. We now document that the absence of Cx40, as well as the interference with this connexin, decreases the formation of new capillary-like vessels, essentially made of ECs, while increasing their recruitment of SMA-positive SMC and stromal cells. Under these conditions, VEGF and its receptor, which are main inducers of angiogenesis [3], were unchanged. In contrast, we document that loss of Cx40 decreased the formation of novel vessels, in association with a reduction of PeNOS, the activated form of the EC enzyme which directly controls the production of NO [28, 29], including in Cx40−/− mice [21]. In turn, this second messenger increases EC differentiation and migration, while inhibiting SMC recruitment [30, 31]. Recent experiments have indicated that Cx40 controls the expression and function of eNOS [15, 21], possibly by decreasing the exchanges of Ca^2+^ transients between EC [16] which modify eNOS activation [32], or by impairing the protein complex that Cx40 forms with the enzyme [33]. Thus, our data implicate that the relationship between Cx40 expression and the production of NO is relevant to the control of tumoral angiogenesis.

Immunofluorescence showed that the labeling of Cx40 within the ECs of these mice was similar to that seen in WT controls. Accordingly, one would expect that the angiogenic parameters we evaluated here would also be alike in Tie2-Cx40 and WT controls, which is what our data indicate. Obviously, the effects which are dependent on Cx40 do not rule out the possible concomitant involvement of the other EC connexins, whether composing gap junction channels or hemi-channels, notably because Cx40 oligomerizes with Cx37 in some connexons [34], and that loss of Cx40 also reduces the expression of Cx37 which has been reported to inhibit Cx40 [15, 21, 35, 36]. Furthermore, our findings do not exclude either that other more intricate mechanisms could contribute to decrease tumoral aniogenesis, including the possible activation of an EC-dependent, local immune reaction triggered after loss of Cx40, and/or other connexin isoforms. Additional mechanisms may be associated with the decreased transfer between ECs of Cx40−/− mice [14] of gap junction-permeant molecules, such as ATP [37], reactive oxygen species, and Ca^2+^ [16, 21, 38], which are all critical mediators for the proper building and function of newly formed vessels. Connexin signaling has been implicated in the control of many secretions, including that of growth factors [39] and renin [9]. By analogy, it is plausible that EC Cx40 may control the secretion of pro-angiogenic molecules, such as platelet-derived growth factor (PDGF), which is necessary for the formation and the maturation of newly formed vessels. In many tumors, the chronic imbalance of pro- and anti-angiogenic signals, notably PDGF, leads to the development of abnormal vasculature [1, 40–42] which, in turn, is thought to impair the access of drugs and antibodies to several types of cancer-cells, presumably contributing to their limited sensitivity to therapy [1]. Thus, preservation of a normal vascular bed, e.g. by altering the tumor environment, is emerging as a necessary complement for improving the efficacy of cytotoxic and radiation therapies [1, 43–45], and it has been proposed that connexins may participate to this improvement [43]. Our data provide evidence that this can be actually achieved in tumors models, since both the loss and inhibition of Cx40 and its targeting by a mimetic peptide markedly extended the survival of tumor-bearing mice, including during a systemic administration of cyclophosphamide.

The findings of such a beneficial effect under different settings (several types of tumors growing in different environments, explant cultures of a normal vessel, *in vivo* implantation of artificial plugs) does not support the view that the connexin effect is dependent on a specific cell type or a given host environment. Rather, our data indicate that the loss of Cx40 signaling consistently decreases the development of neo-vessels, while improving their maturation, as judged by the increased coverage of ECs by SMA-expressing contractile cells. Strikingly, this connexin-dependent effect was evident under conditions of actively stimulated angiogenesis in both tumoral and non tumoral tissues, but was not observed under the more chronic, steady state conditions of control vessels renewal, which was presumably the situation in the histologically normal regions of the murine bladders we analyzed. If validated, this differential effect could largely benefit the future therapeutic translation of the experimental approach we describe here. Indeed, since the vessels of human tumors express Cx40 (Supplementary Figure S6), a protein which shares large homology with mouse Cx40 in the extra-cellular region which is targeted by ^40^Gap27 [46], and since loss of Cx40 does not appear to significantly alter the microcirculation established in normal adult tissues, our findings open the challenging perspective that targeting Cx40 may also be useful in the treatment of cancer patients.

## MATERIALS AND METHODS

### Animals and human samples

8–12 weeks-old female C57BL/6 wild type (WT), Cx40−/− [47] and Tie2-Cx40 mice expressing Cx40 solely in ECs [21], were used according to the animal procedures approved by our institutional and state committees for animal experiments, and conform to the Guide for the Care and Use of Laboratory Animals (US National Institutes of Health, 8th edition, 2011). All transgenic mice were maintained on a C57BL/6 background and genotyped by PCR [15, 21].

The generation of Cx40−/− and Tie2-Cx40 mice and their genotypic characterization has been described [17, 35]. The Tie2-Cx40 mice used in this study, were generated by crossing Tie2-Cx40 heterozygous transgenics with C57BL/6-Cx40−/− mice [21]. We have previously reported that these animals express Cx40 only in ECs, at levels that, in individual cells assessed by immunofluorescence, appeared alike those of WT controls, still in tissue extracts assessed by Western blotting, were overall intermediate between those of Cx40−/− and WT control mice, presumably because Cx40 expression was not induced in all ECs [21].

In some experiments, WT and Cx40−/− mice received for 3 weeks drinking water containing 3 mg/kg. day Candesartan (Cilexetil^®^, Atacand, Astra Zeneca), and blood pressure was monitored by tail cuff plethysmography method as previously described [21, 35, 48]. Samples of invasive bladder cancers were obtained at cystectomies after informed patient consent and with the authorization of our state ethical committee (study 119/10).

### Tumor models

Two widely used subcutaneous tumor models were generated in WT, Cx40−/− and Tie2-Cx40 mice using TC-1 cells (human papillomavirus oncogene expressing cells derived from C57/Bl6 lung epithelium [49] or B16-F10 mouse melanoma cells [50]. Mice were (s.c.) injected into the flank with 2 × 10^4^ TC-1 or 2.5 × 10^5^ B16-F10 cells and tumor-growth monitored every two days with a caliper. Tumor volume was calculated as length × width^2^ /2 and mice sacrificed when tumor volumes were > 1.25 cm^3^, according to veterinary guidelines. For comparison to healthy organs, orthotopic bladder tumors were also generated by intravesically instilling 2.5 × 10^5^ TC-1 cells into the bladder, as previously described [51]. The size of these tumors was estimated by weighing the bladders at animal sacrifice. At that time, all tumors were frozen into liquid nitrogen.

In some experiments, 50 mg/kg b.w. cyclophosphamide (CTX, C-0768 from Sigma), were intraperitoneal (i.p.) injected three times, once a week, for 3 weeks (at days 8, 15 and 22) into subcutaneous TC-1 tumor-bearing mice [52]. In other experiments, WT mice were also i.p. injected every day with 100 μg of either ^40^Gap27 peptide or its scrambled version, starting one day after tumor cell implantation. The sequence of the ^40^Gap27 peptide differs by 3 amino acids of the fourth transmembrane connexin stretch from that of peptide Gap27, which was originally generated to inhibit Cx43 [22]. Previous studies have documented that ^40^Gap27 does not inhibit Cx43 but specifically targets Cx40 [23, 25]. The concentrations of the ^40^Gap27 peptide tested in this study were similar to those reported to be effective in blocking Cx40 function in previous studies [22, 23, 25].

### Matrigel plug assay

Plugs of 0.5 mL Growth Factor Reduced matrigel (BD Biosciences, 356231) supplemented with either 500 ng/ml recombinant murine FGF-2 (Peprotech, 450–33) and 3 U/ml Heparin (Biochrom AG, L6510) or 200 ng/ml recombinant murine VEGF_165_ (Peprotech, 450–32) and 10 U/ml Heparin [53], were s.c. injected into the flank of either WT or Cx40−/− mice. Matrigel lacking growth factors were used as negative control. WT mice were also injected with Growth Factor Reduced matrigel supplemented with 200 ng/ml VEGF, 10 U/ml Heparin and 300 μM of either peptide ^40^Gap27 or its scrambled version. The plugs were removed 1 week after injection, for evaluation of vascularization.

### Aortic ring assay

Mouse aortas from 10 week-old WT and Cx40−/− mice, were cut into rings of 1 mm thickness [54], dropped in 20 μl matrigel previously polymerized during 30 min at 37°C (BD Biosciences, 356237), and overlaid with 30 μl matrigel (BD Biosciences, 356237). After another 30 min polymerization at 37°C, Endothelial Cell Growth Medium-2 (Lonza, EGM^™^-2 BulletKit^™^, C2517A) was added, and then changed every 3 days. In some experiments, the assay was performed on aortic rings from mice pretreated for 1 week with 100 μg of either peptide ^40^Gap27 [23] or a scrambled version of this peptide (Protein and Peptide Chemistry Facility of the Institute of Biochemistry, UNIL, Switzerland), and given by intraperitoneal (i.p.) injection every two days. Aortic rings were then embedded in matrigel supplemented with 300 μM either peptide ^40^Gap27 [23] or the scrambled peptide. In all cases, vessel sprouts were photographed with a bright-field contrast microscope (Nikon Eclipse TS100), from day 3 to 10.

### Immunostaining

Ethanol-fixed aortic rings and 8 μm-thick cryosections of tissues and tumors were incubated in blocking buffer (PBS, 2% BSA, 0.3% Triton X-100) for 1 h at room temperature, and then incubated overnight at 4°C in PBS with one of the following antibodies against: CD31 (BD Pharmingen, 553371), diluted 1:100; Cx40 (Alpha Diagnostic International, Cx40-A), diluted 1:50; cleaved VEGFR2 (Cell Signaling Technology, 2479), diluted 1:100; Von Willebrand Factor (VW, Dako, A0082), diluted 1:500; Phospho eNOS (PeNOS, Cell Signaling Technology, 9571), diluted 1:100; alpha-smooth muscle actin (SMA, Abcam, ab5694) diluted 1:100. Samples were then incubated for 2 h at room temperature with either Alexa-Fluor-594- or -488-coupled secondary antibodies (Life Technologies), diluted 1:500 in PBS, covered with PBS containing 50% glycerol and 0.4 μg/mL DAPI, and observed by fluorescence microscopy (Leica Leitz DMRB, Nidau, Switzerland). Frozen sections from human bladder biopsies were also stained with hematoxylin and eosin.

### Hemoglobin content

Hemoglobin content was quantified using the Drabkin's reagent kit (Sigma Chemie, Buchs Switzerland). Briefly, 100 mg tumor powder or excised matrigel-plugs were homogenized in 0.5 mL 0.1% Brij-35 lysis buffer, and centrifuged for 5 min at 10,000 g. 50 μL of the supernatant was mixed with 450 μL Drabkin's reagent, and absorbance read at 540 nm. The concentration of hemoglobin was calculated from a cyan-methemoglobin standard.

### Tumor perfusion assay

Mice were intravenously (i.v.) injected with 100 μg FITC-tomato lectin (Vector, FL-1171, diluted in PBS). Five min later, mice were perfused systemically with 4% paraformaldehyde through the left heart ventricle, and then sacrificed before tumor sampling. Tumor tissues were fixed in 4% paraformaldehyde for 2–3 additional hours, and incubated in 30% sucrose overnight at 4°C. The tissues were then embedded in OCT compound, cut at 20 μm thickness and kept frozen at −80°C. CD31 was immunostained as described above. Cell nuclei were counterstained with DAPI, and sections observed by fluorescence microscopy.

### *In vitro* angiogenesis and SMC recruitment assays

For *in vitro* angiogenesis assays, Matrigel (BD Biosciences, 356231) was coated onto 24-well culture plates and polymerized for 15 min at 37°C. HUVECs (1 × 10^5^ cells/well) were seeded on Matrigel and incubated at 37°C for 12 h. To evaluate the effects of Cx40 inhibition on this process, similar experiments were conducted in presence of 300 μM ^40^Gap27 peptide [23, 25] or its scrambled version directly added to the growth medium. Capillary-like structures were observed and photographed through an inverted microscope Nikon TS100 (Nikon). Quantification of capillary-like structures was performed using the Angiogenesis analyzer plug-in on ImageJ software [26]. For SMC recruitment assays, the rat aortic VSMC line A7r5 obtained from American Type Culture Collection (ATCC; Manassas, VA, USA) was used. A7r5 cells were maintained in Dulbecco's modified Eagle's low glucose medium (DMEM; 31885–023, Life Technologies) supplemented with 10% fetal calf serum (FCS) and 1% penicillin/streptomycin at 37°C; 5% CO2 and labeled with the PKH67 Green Fluorescent Cell Linker Kit (Sigma) according to manufacturer's instructions. Fluorescent A7r5 cells (1 × 10^5^ cells/well) were seeded into wells containing HUVECs capillary-like structures generated under control conditions or after addition of 300 μM ^40^Gap27 peptide or its scrambled version to the media. Motility of SMCs was assessed by time-lapse microscopy using a Cytation3 Cell Imaging Multi-Mode Reader (BioTek). Images were acquired every 5 minutes over 6–8 hours. The quantification of SMC recruitment to capillary-like structures after 6 h was performed using ImageJ software.

### Protein analysis

100 μg powder of s.c. B16-F10 tumors or confluent HUVECs collected from a 12-well plate were homogenized by sonication in SDS Lysis Buffer (62.5 mM Tris-EDTA, pH 6.8, 5% SDS). Protein content was measured using a detergent-compatible protein assay kit (Bio-Rad Laboratories, Reinach BL, Switzerland). 25 μg proteins were loaded on a 10% polyacrylamide gel, electrophoresed and transferred onto PVDF membrane (Immobilon-P; Millipore, Volketswil, Switzerland). Membranes were incubated for 1 h in TBS containing 5% milk and 0.1% Tween20 (blocking buffer). Saturated membranes were incubated overnight at 4°C with the following antibodies, against: Von Willebrand factor (Dako, A0082), diluted 1:2000; Phospho eNOS (PeNOS, Cell Signaling Technology, 9571), diluted 1:1000; eNOS (BD Biosciences, 610297), diluted 1:1000; VEGFR2 (Cell Signaling Technology, 2479), diluted 1:1000; MMP2 (Abcam, ab37150), diluted 1:1000; VE-cadherin (Santa Cruz Biotechnology, sc-6458), diluted 1:1000; smooth muscle actin (SMA, Abcam, ab5694) diluted 1:1000 or monoclonal antibody anti-alpha-tubulin (T5168, Sigma-Aldrich, 1:3000). Membranes were then incubated at room temperature for 1 h with a convenient secondary antibody conjugated to horseradish peroxidase (FlukaChemie, diluted 1:20,000). Bands were developed using enhanced chemiluminescence (Millipore, Immunobilon Western Chemiluminescent HRP substrate), and visualized using a supercooled CCD camera (Chemidoc XRS, Bio-Rad Laboratories). Densitometric analysis was performed using ImageLab Software (3.0.1 Bio-Rad Laboratories).

### Statistical analysis

All tissue staining measurements were made using the ImageJ software (NIH Image, Bethesda, MD). Statistical analyses were performed using Prism 6.00 for Windows (GraphPad software, San Diego, Ca). Differences between groups were performed by Mann-Whitney test or by Student *t* test whichever applicable. Multiple comparisons were performed using one-way ANOVA and Tukey's Multiple Comparison Test. Other statistical tests are indicated in the text or figure legends.

## SUPPLEMENTARY MATERIALS FIGURES


