# Supplementary material for: Targeting endothelial connexin40 inhibits tumor growth by reducing angiogenesis and improving vessel perfusion
Source: Oncotarget. 2016 Feb 13;7(12):14015–28. doi: 10.18632/oncotarget.7370 (PMC4924695; doi:10.18632/oncotarget.7370)
Supplement: Supplementary file 1 [file oncotarget-07-14015-s001.pdf]

Targeting endothelial connexin40 inhibits tumor growth by reducing angiogenesis and improving vessel perfusion

Supplementary Materials

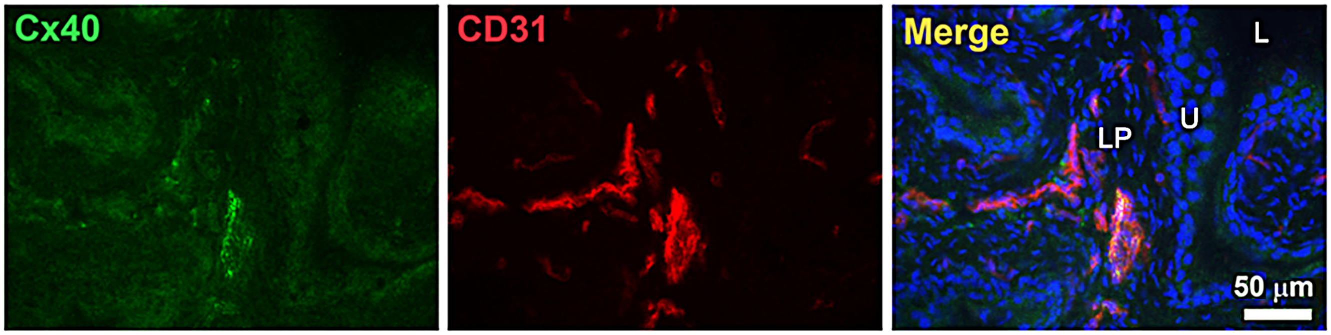

Supplementary Figure S1: Cx40 is expressed within blood vessels located in the lamina propria of WT native bladders. Immunostaining of the native bladder of a WT mouse shows that the Cx40 staining colocalizes with that of CD31, a marker of ECs.

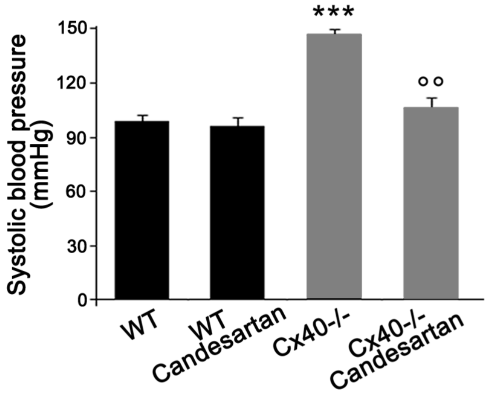

Supplementary Figure S2: Systolic blood pressure of WT and Cx40–/– mice following treatment with Candesartan. A 3 week-long treatment with Candesartan did not alter the systolic blood pressure of WT mice, but significantly reduced the hypertension of Cx40–/– mice, Data are means + SEM of 8 mice per group. ***p < 0.001 versus untreated WT mice; °°p < 0.01 versus untreated Cx40–/–mice.

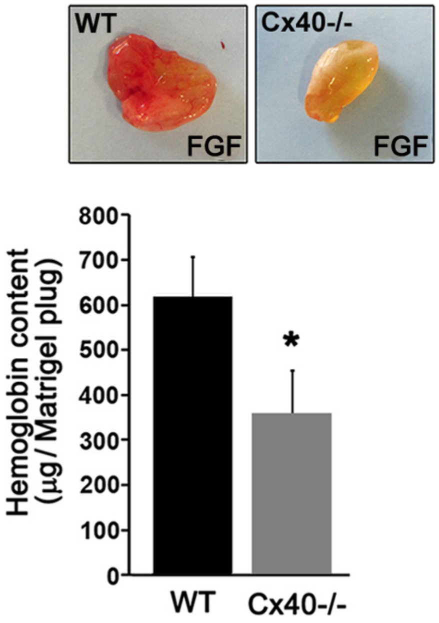

Supplementary Figure S3: Hemoglobin content is decreased in FGF supplemented matrigel plugs in Cx40–/– mice. One week after the s.c. injection in WT mice, plugs supplemented with FGF were intensively stained in red and featured sizable hemoglobin content, indicating an important colonization by angiogenic vessels. Both the red color and the hemoglobin content were reduced in plugs retrieved from Cx40–/– mice. Data are means + SEM of 11–13 mice per group. *$p < 0.05$ versus WT mice.

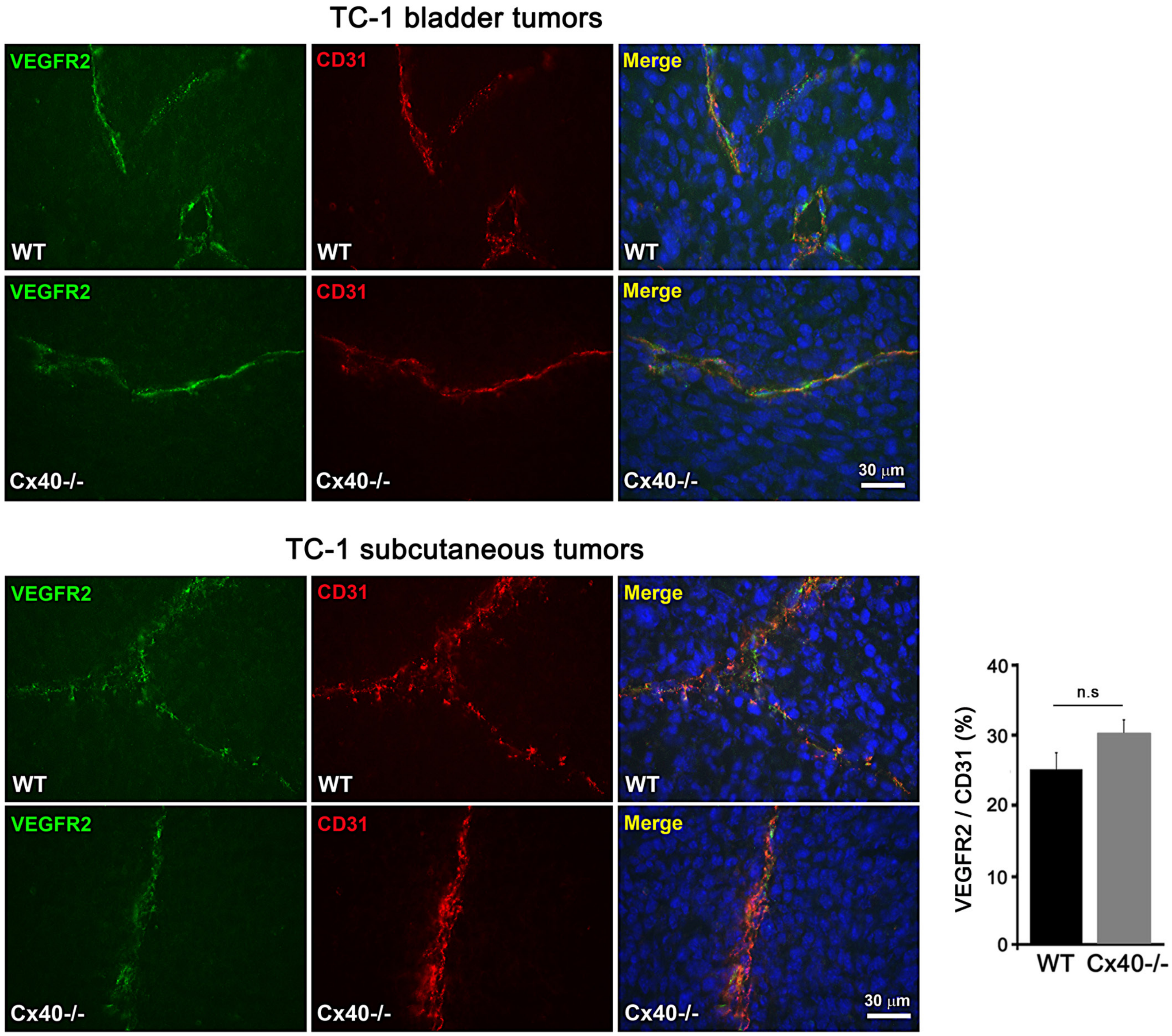

Supplementary Figure S4: VEGFR2 expression in blood vessels from bladder and s.c tumors is not altered by Cx40 deficiency. Double-staining of the specific endothelial cells marker CD31 (red) with VEGFR2 (green) performed on cross-sections of bladder and subcutaneous tumors from WT and Cx40–/– mice. Data are mean + SEM of 2 fields from 3 different mice per group.

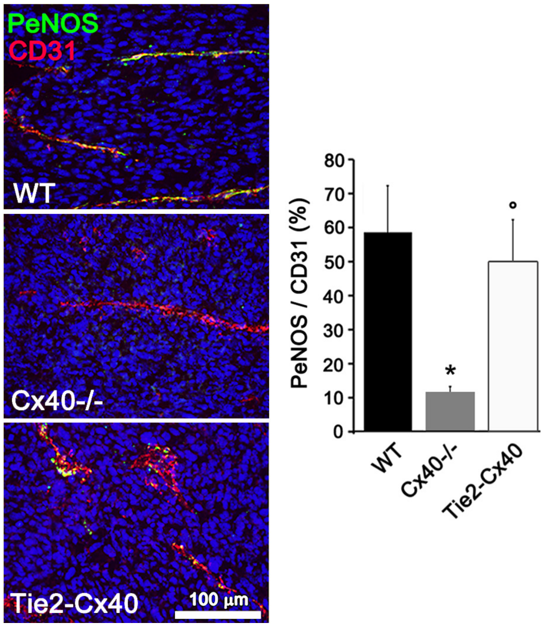

Supplementary Figure S5: The phosphorylation of eNOS is decreased in vessels of TC-1 tumors. Immunostaining showed the presence of PeNOS on CD31 positive ECs of all TC-1 tumors from WT mice. The density of the PeNOS staining was similar in the tumors induced in Tie2-Cx40 and WT mice; but significantly lower in the tumors induced in Cx40–/– mice. Data are mean + SEM of 3–8 fields from 4–5 different mice per group. Significant differences are shown as $*p < 0.05$ versus untreated WT mice; $°°p < 0.05$ versus untreated Cx40–/–mice.

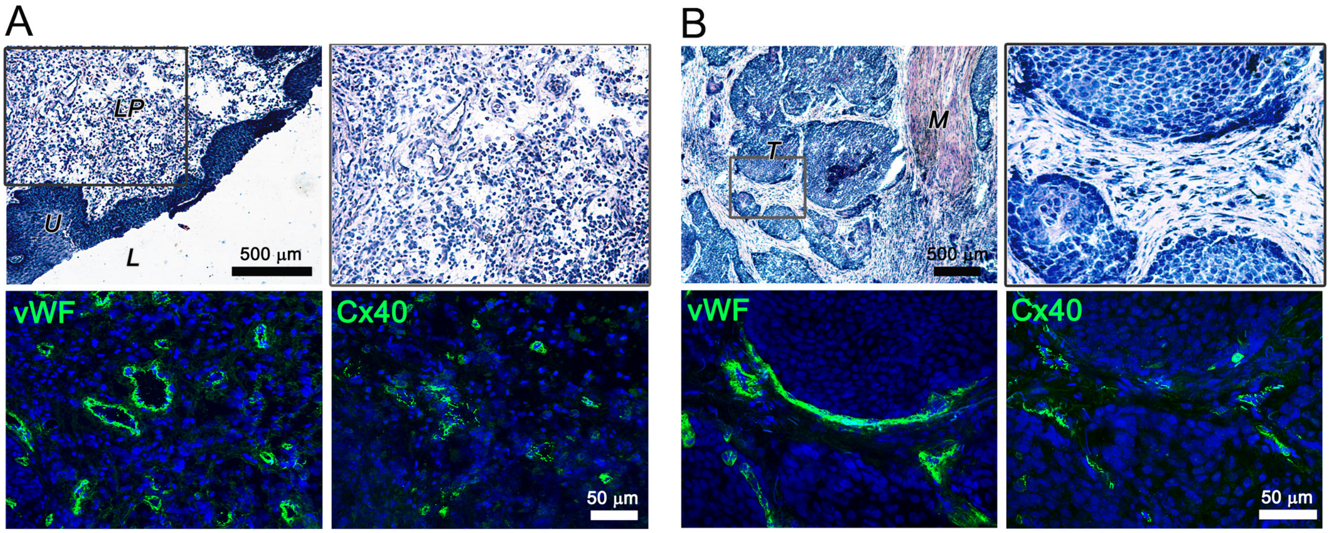

Supplementary Figure S6: Cx40 is expressed in the vessel of human bladder cancer. Immunostaining of a surgical cystectomy specimen revealed the presence of Cx40 in the vessels of both a non-invaded region of the bladder (**A**) and of an adjacent invasive tumor mass (**B**). vWF: von Willebrand Factor LP: lamina propria; U: urothelium; L: lumen; T: tumor; M: muscle.
